# Exaggerated exercise pressor reflex in male UC Davis type 2 diabetic rats is due to the pathophysiology of the disease and not aging

**DOI:** 10.3389/fphys.2022.1063326

**Published:** 2023-01-10

**Authors:** Yu Huo, Ann-Katrin Grotle, Richard K. McCuller, Milena Samora, Kimber L. Stanhope, Peter J. Havel, Michelle L. Harrison, Audrey J. Stone

**Affiliations:** ^1^ Department of Kinesiology and Health Education, The University of Texas at Austin, Austin, TX, United States; ^2^ Department of Sport, Food and Natural Sciences, Western Norway University of Applied Science, Bergen, Norway; ^3^ Department of Molecular Biosciences, School of Veterinary Medicine and Department of Nutrition, University of California, Davis, Davis, CA, United States

**Keywords:** autonomic control of circulation, blood pressure, mechanoreflex, metaboreflex, cardiovascular response

## Abstract

**Introduction:** Studies in humans and animals have found that type 2 diabetes mellitus (T2DM) exaggerates the blood pressure (BP) response to exercise, which increases the risk of adverse cardiovascular events such as heart attack and stroke. T2DM is a chronic disease that, without appropriate management, progresses in severity as individuals grow older. Thus, it is possible that aging may also exaggerate the BP response to exercise. Therefore, the purpose of the current study was to determine the effect of the pathophysiology of T2DM on the exercise pressor reflex independent of aging.

**Methods:** We compared changes in peak pressor (mean arterial pressure; ΔMAP), BP index (ΔBPi), heart rate (ΔHR), and HR index (ΔHRi) responses to static contraction, intermittent contraction, and tendon stretch in UCD-T2DM rats to those of healthy, age-matched Sprague Dawley rats at three different stages of the disease.

**Results:** We found that the ΔMAP, ΔBPi, ΔHR, and ΔHRi responses to static contraction were significantly higher in T2DM rats (ΔMAP: 29 ± 4 mmHg; ΔBPi: 588 ± 51 mmHg•s; ΔHR: 22 ± 5 bpm; ΔHRi: 478 ± 45 bpm•s) compared to controls (ΔMAP: 10 ± 1 mmHg, *p* < 0.0001; ΔBPi: 121 ± 19 mmHg•s, *p* < 0.0001; ΔHR: 5 ± 2 bpm, *p* = 0.01; ΔHRi: 92 ± 19 bpm•s, *p* < 0.0001) shortly after diabetes onset. Likewise, the ΔMAP, ΔBPi, and ΔHRi to tendon stretch were significantly higher in T2DM rats (ΔMAP: 33 ± 7 mmHg; ΔBPi: 697 ± 70 mmHg•s; ΔHRi: 496 ± 51 bpm•s) compared to controls (ΔMAP: 12 ± 5 mmHg, *p* = 0.002; ΔBPi: 186 ± 30 mmHg•s, *p* < 0.0001; ΔHRi: 144 ± 33 bpm•s, *p* < 0.0001) shortly after diabetes onset. The ΔBPi and ΔHRi, but not ΔMAP, to intermittent contraction was significantly higher in T2DM rats (ΔBPi: 543 ± 42 mmHg•s; ΔHRi: 453 ± 53 bpm•s) compared to controls (ΔBPi: 140 ± 16 mmHg•s, *p* < 0.0001; ΔHRi: 108 ± 22 bpm•s, *p* = 0.0002) shortly after diabetes onset.

**Discussion:** Our findings suggest that the exaggerated exercise pressor reflex and mechanoreflex seen in T2DM are due to the pathophysiology of the disease and not aging.

## Introduction

Individuals with type 2 diabetes mellitus (T2DM) have an increased risk of cardiovascular diseases, such as coronary heart disease and atherosclerosis (Pop-Busui et al., 2017; La Sala et al., 2019; Lu et al., 2019), which contributes to a 3-fold greater risk of premature death (Sattar et al., 2019). Individuals with T2DM are commonly prescribed regular exercise or physical activity to improve glucose control and cardiovascular health (Stewart, 2002). However, human and animal studies have shown that acute exercise evokes exaggerated blood pressure (BP) and heart rate (HR) responses in those with T2DM (Scott et al., 2008; Holwerda et al., 2016; Grotle et al., 2019; Kim et al., 2019). Importantly, these exaggerated cardiovascular responses may increase the risk of adverse cardiovascular events, such as myocardial infarction and stroke. The mechanisms responsible for the exaggerated BP and HR responses to exercise in T2DM are not fully understood; however, recent studies suggest the exercise pressor reflex likely plays an important role (Holwerda et al., 2016; Grotle et al., 2019; Kim et al., 2019; Vranish et al., 2020).

The exercise pressor reflex is a feedback mechanism that regulates BP and HR during exercise (Alam and Smirk, 1937; McCloskey and Mitchell, 1972). The sensory arm of the reflex comprises a mechanically sensitive component conducted predominantly through thinly myelinated group III muscle afferents, namely the mechanoreflex; and a metabolically sensitive component conducted predominantly through unmyelinated group IV muscle afferents, namely the metaboreflex (Kaufman et al., 1983). Research has shown that both the mechanoreflex and the metaboreflex are exaggerated in T2DM (Holwerda et al., 2016; Grotle et al., 2019; Kim et al., 2019; Ishizawa et al., 2021). For example, passive tendon stretch, which activates the mechanoreflex, evoked significantly higher BP and HR responses in T2DM rats compared to their healthy control (Grotle et al., 2019). In addition, the pressor, cardioaccelerator, and sympathetic responses are significantly higher in response to capsaicin, which activates the metaboreflex, in T2DM rats compared to their healthy controls (Ishizawa et al., 2021). These studies strongly support that the exercise pressor reflex is exaggerated in T2DM (Holwerda et al., 2016; Grotle et al., 2019; Kim et al., 2019; Vranish et al., 2020).

A previous study from our lab using the University of California Davis (UCD) T2DM rat model found that the pressor responses to muscle contraction and tendon stretch are not consistently exaggerated throughout the duration of the disease; however, the effects of aging were not accounted for (Grotle et al., 2019). Previous findings on the effect of aging on the pressor responses to exercise are inconclusive. Several studies have reported that the BP response to exercise was significantly higher in older adults compared to younger adults (Hasegawa et al., 2021; O'Connor et al., 2015; Petrofsky and Lind, 1975). Whereas other studies reported similar or even lower BP responses to exercise in older adults compared to young adults (Sagiv et al., 1988; Taylor et al., 1991; Markel et al., 2003). Furthermore, it is well established that sympathetic nerve activity is increased at rest and during exercise with aging (Rowe and Troen, 1980; Esler et al., 1995), which could evoke exaggerated BP and HR responses. Therefore, it is possible that the exaggerated cardiovascular responses observed in T2DM rats were partially due to the aging process that occurs concurrent with the changing pathophysiology of the disease.

Therefore, the purpose of the current study was to determine the effect of T2DM on the exercise pressor reflex independent of aging. We tested the hypothesis that the exaggerated exercise pressor reflex seen in T2DM is due to the pathophysiology of the disease, and not aging, by assessing the pressor responses to muscle contraction and tendon stretch in UCD-T2DM rats compared to healthy, age-matched controls throughout the course of the disease.

## Materials and methods

All procedures were reviewed and approved by the Institutional Animal Care and Use Committee of The University of Texas at Austin. Adult male (*n* = 30) UCD-T2DM rats from the breeding colony in the Department of Nutrition at UCD were used. The UCD-T2DM rat model combines polygenic adult-onset obesity and insulin resistance from obese Sprague Dawley rats with inadequate islet function/β-cell compensation from Zucker diabetic fatty-lean rats resulting in overt diabetes with a progressive increase in fasting and non-fasting plasma glucose concentrations and elevated HbA1c levels (Cummings et al., 2008). As a result, rats develop T2DM over time with a pathophysiology similar to that in humans. Female rats from this cohort were much older than male rats when they developed diabetes, which inhibited our ability to track their progression over time as they aged. Therefore, only data from male rats is presented in this study, and this is recognized as a limitation of our findings. We measured the pressor responses at three different stages during disease progression: 1) early-onset: 7–15 weeks after the onset of T2DM; 2) established: 29–31 weeks after the onset of T2DM; and 3) chronic: 43–46 weeks after the onset of T2DM. It is important to note that the three stages are simply different snapshots of the pathophysiology, and do not represent specific thresholds of the disease. We compared these responses to healthy, age-matched male Sprague Dawley rats (*n* = 32) (Charles River, Wilmington, MA), as Sprague Dawley are ideal controls for the UCD-T2DM rats as they age naturally without any cardiovascular diseases (Capitanio et al., 2016). T2DM was diagnosed using fasting blood glucose (STAT STRIP Xpress, Nova Biomedical, Waltham, MA). The rats were housed in a temperature-controlled room (24°C ± 1°C) with a 12:12 h light-dark cycle and fed a standard diet and tap water *ad libitum*. After terminal experiments, rats were euthanized by ventilation with 5% isoflurane and injected intravenously with saturated potassium chloride (>3 ml/kg), followed by a thoracotomy.

### Blood sampling and insulin measurement

Blood samples were drawn from the tail vein three to 7 days before the experiments. Blood samples were left to clot for 30 min before being centrifuged for 10 min at 1400 × g. Serum was then aliquoted into tubes and frozen at −80°C for subsequent insulin analyses. An ultrasensitive rat insulin ELISA kit (ALPCO (80-INSRTU-E01), Salem, NH) was used to measure insulin concentrations in the samples. Standards, controls, and samples were added, in duplicate, to a 96-well plate and analyzed according to the manufacturer’s recommendations. Plates were read using an infinite F200 pro microplate reader (TECAN, Switzerland) to determine the absorbance for each well. Four-parameter-Marquardt logistic regression was used to construct a standard curve, which was then used to calculate control and sample concentrations. *R* ^2^ for the standard curve was 0.999, and the coefficient of variation between sample duplicates averaged <12.2%.

### Surgical preparation

On the day of the experiment, overnight fasted blood glucose (STAT STRIP Xpress, Nova Biomedical, Waltham, MA) and hemoglobin A1c (HbA1c) (A1CNow^+^, PTS diagnostics, Indianapolis, IN) were assessed by pricking the tail of the rats. The rats were then anesthetized with isoflurane gas (2%–5%) in 100% oxygen, and body weights were assessed. We cannulated the trachea to mechanically ventilate the lungs (Model 683 small animal ventilator, Harvard Apparatus, Holliston, Massachusetts). We cannulated the right jugular vein and both common carotid arteries (PE-50) for fluid and drug delivery and BP measurement, respectively. Although both carotid arteries were cannulated, the baroreflex remained intact during the surgical preparation, as demonstrated by previous studies (Copp et al., 2016). One of the carotid arterial catheters was connected to a pressure transducer (CWE DTX-1, Ardmore, PA) for measuring BP and HR (CED, Cambridge, UK), and the other catheter was used for analyzing blood gases (Nova Biomedical, Waltham, MA).

The left hindlimb muscles were exposed, and the skin was retracted. We surgically isolated the sciatic nerve and placed a stimulating electrode underneath the sciatic nerve to evoke muscle contractions. The calcaneal bone of the same leg was severed, and the Achilles tendon was attached to a force transducer (model FT-03, Grass Instruments, West Warwick, RI), which was connected to a rack-and-pinion for tension measurement. We used an automated blood gas analyzer to measure arterial PO_2_, PCO_2_, and pH. Arterial blood gas and pH were maintained within normal range by either adjusting ventilation or intravenously injecting sodium bicarbonate (8.5%). A rectal temperature probe was used to measure body temperature, which was maintained between 36.5°C and 38°C using a heating lamp and plate.

Rats were then placed in a Kopf stereotaxic frame for the decerebration procedure; the spine and pelvis of the rat were stabilized using a pair of metal spikes. Dexamethasone (2 mg/ml; 0.2 ml) was injected intravenously to prevent excessive swelling in the brain before performing a pre-collicular decerebration. All neural tissue rostral to the section was removed, and the cranial vault was filled with gauze to stop bleeding. Immediately after the decerebration, we discontinued the gas anesthesia, allowing rats to stabilize for at least 1 h before starting the experimental protocols (Smith et al., 2001).

### Static contraction

Before contraction, the Achilles tendon was stretched to set a baseline tension (90–100 g) for 30 s. We then evoked a static contraction by electrically stimulating (40 Hz; 0.01 ms; ≤2 times motor threshold) the sciatic nerve for 30 s and, during the stimulation, measured mean arterial pressure (MAP), HR, and tension time index (TTI). This protocol simulates static muscle contraction, similar to isometric exercise.

### Intermittent contraction

Before contraction, the Achilles tendon was stretched to set a baseline tension (90–100 g) for 30 s. We then evoked an intermittent contraction by electrically stimulating (40 Hz, 0.01 ms pulse duration, 500 ms train duration, ≤2 times motor threshold) the sciatic nerve for 30 s and, during the stimulation, measured MAP, HR, and TTI. This protocol simulates dynamic muscle contraction, similar to rhythmic dynamic exercise.

### Tendon stretch

Before the passive tendon stretch, the Achilles tendon was stretched to set a baseline tension (90–100 g) for 30 s. We then stretched the Achilles tendon by rapidly turning the rack-and-pinion, and a tension equivalent to that achieved during static muscle contraction was maintained for 30 s and, throughout the stretch, MAP, HR, and TTI were measured.

To verify that the pressor responses during muscle contractions were not due to direct stimulation of the skeletal muscle afferents that are responsible for evoking the exercise pressor reflex, we injected 0.5 ml of pancuronium bromide (1 mg/ml; Sigma Aldrich, St. Louis, MO) through the jugular vein to paralyze the rat. Then, we stimulated the sciatic nerve again using the same parameters as those described above. If pancuronium bromide abolished the pressor response to electrical stimulation of the sciatic nerve, we concluded that the pressor response to muscle contraction was not due to any direct stimulation of skeletal muscle afferents.

### Data analysis

We present all data as means ± SE, as well as individual data points. Real-time MAP (mmHg), HR (bpm), and muscle tension (g) were recorded with a Spike2 data acquisition system (Cambridge Electronic Design). The peak pressor and cardioaccelerator responses were recorded for one complete cardiac cycle regardless of where they occurred during the 30 s muscle contraction. The second-by-second time course of the pressor and cardioaccelerator responses to muscle contraction were expressed as their change from baseline (∆). The BP-time index (BPi, mmHg·s), HR-time index (HRi, bpm·s), and TTI (kg·s) were calculated by subtracting the integrated area under each respective trace during the 30 s baseline period from the integrated area under the trace for the 30 s contraction period. The glucose and HbA1c data were tested with one tailed student’s t-test within each stage. The body weight, triceps surae weight, insulin, baseline MAP and HR, and TTI were tested with two tailed student’s t-test within each stage. Changes in MAP, HR, and BPi were compared using two-way ANOVAs. Holm-Sidak’s *post hoc* tests were used when a significant interaction was detected. We conducted all statistical analyses with Prism 8-GraphPad Software (La Jolla, CA). The criterion level of significance was set at *p* < 0.05.

## Results

### Baseline measurements

Body weight, blood glucose, HbA1c, and insulin concentrations for all groups are shown in Table 1. Body and triceps surae muscle weights of T2DM rats were significantly lower compared to healthy, age-matched controls (all *p* < 0.05) at each stage of T2DM. As expected, the glucose and HbA1c levels were significantly higher in T2DM rats compared to healthy, age-matched rats at all stages of T2DM (all *p* < 0.05). Conversely, serum insulin concentration was significantly lower in T2DM rats compared to healthy, age-matched controls during the early onset stage (T2DM: *n* = 9; healthy: *n* = 8; *p* = 0.03) and the chronic stage (T2DM: *n* = 9; healthy: *n* = 7; *p* = 0.001), but not the established stage (T2DM: *n* = 10; healthy: *n* = 10; *p* = 0.11).

**TABLE 1:** Characteristics of healthy and T2DM rats at different stages of the disease.

Stages	Early onset		Established		Chronic	
Groups	Healthy	T2DM	Healthy	T2DM	Healthy	T2DM
Body weight (g)	689 ± 14	511 ± 13*	714 ± 44	475 ± 25*	594 ± 35	461 ± 24*
Triceps Muscle weight (g)	4.6 ± 0.1	3.4 ± 0.1*	3.7 ± 0.2	3.0 ± 0.2*	3.5 ± 0.1	2.7 ± 0.1*
Fasted blood glucose (mg/dl)	81 ± 5	436 ± 32*	88 ± 3	366 ± 51*	88 ± 4	378 ± 50*
HbA1c (%)	4.4 ± 0.1	12.9 ± 0.1*	4.7 ± 0.4	12.2 ± 0.4*	4.5 ± 0.1	12.5 ± 0.3*
Insulin (ng/ml)	0.64 ± 0.13	0.33 ± 0.05*	1.15 ± 0.43	0.41 ± 0.11	0.77 ± 0.10	0.31 ± 0.06*

Values are means ± SE; Student’s one tailed *t*-test for glucose and HbA1c group comparisons within each stage; Student’s two tailed *t*-test for body weight, triceps muscle weight, and insulin group comparisons within each stage. (*) significantly different from the age-matched healthy rats, *p* < 0.05.

### Static muscle contraction

**FIGURE 1:**
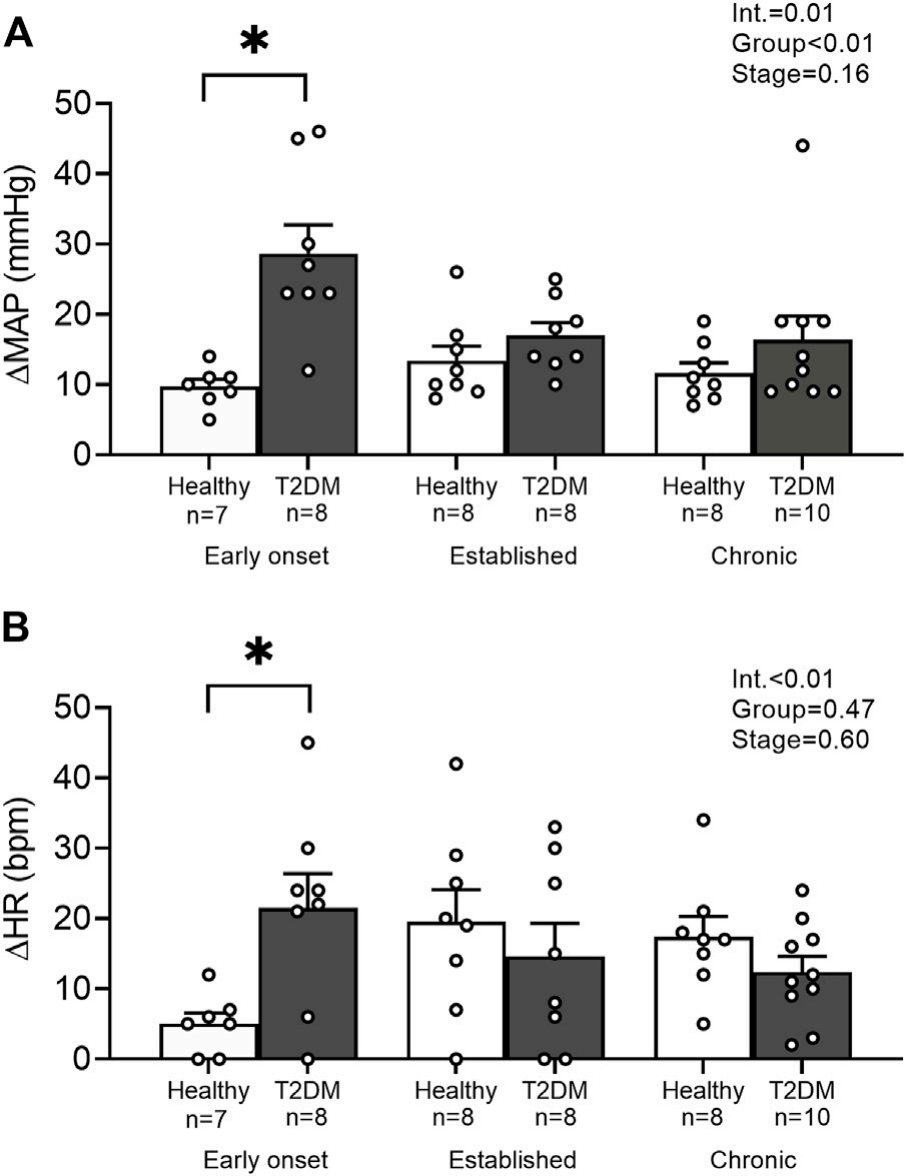
Means ± SE and individual data points showing that statically contracting the hindlimb muscles evoked exaggerated peak pressor **(A)** and cardioaccelerator responses **(B)** during the early onset stage in male T2DM rats compared to healthy, age-matched controls. Two-way ANOVA with Holm-Sidak’s *post hoc* comparisons (*) *p* < 0.05 indicates significant increase relative to age-matched control.

**FIGURE 2:**
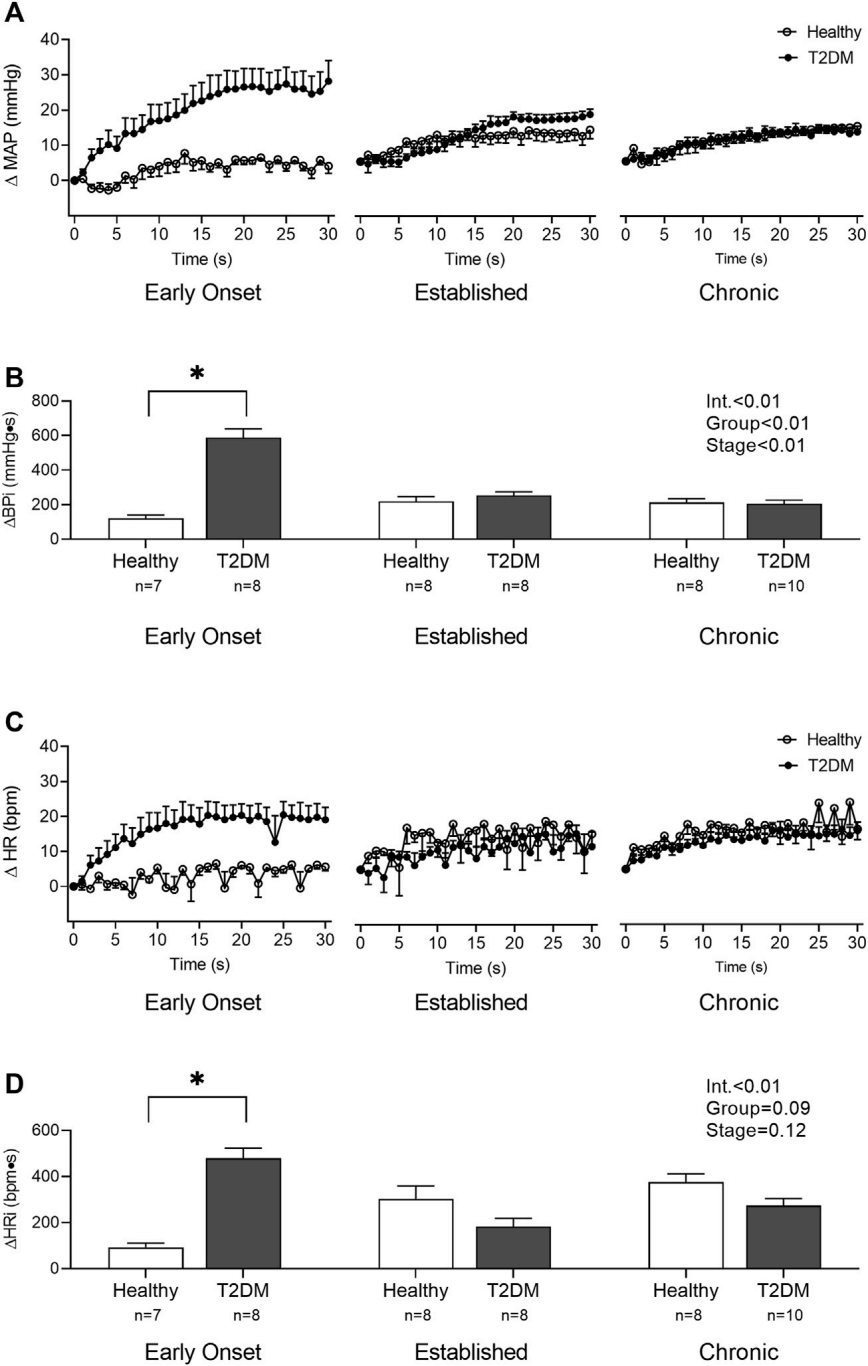
The change in MAP **(A)** and HR **(C)** baseline were averaged second by second to show the temporal changes during 30 s of static contraction in male T2DM rats and healthy, age-matched controls. ΔBPi **(B)** and ΔHRi **(D)** were then calculated over the 30 s of contraction to determine the difference between T2DM rats and healthy, age-matched controls at all stages. The ΔBPi and ΔHRi were greater in the T2DM rats compared to healthy, age-matched controls during the early onset stage of T2DM. Two-way ANOVA with Holm-Sidak’s *post hoc* comparisons (*) *p* < 0.05 indicates significant increase relative to age-matched control.

Baseline MAP and HR were similar in all groups (Table 2, *p* > 0.05), except baseline HR between T2DM rats and their healthy, age-matched controls during the early onset stage, *p* = 0.01. The peak pressor (*p* < 0.01, Figure 1A) and cardioaccelerator (*p* = 0.01, Figure 1B) responses to static contraction were significantly higher in T2DM rats during the early onset stage compared to healthy, age-matched controls. However, neither the pressor nor the cardioaccelerator responses were significantly different in T2DM rats compared to healthy, age-matched controls during the established stage (ΔMAP: *p* = 0.36, Figure 1A; ΔHR: *p* = 0.74; Figure 1B) or the chronic stage (ΔMAP: *p* = 0.36, Figure 1A; ΔHR: *p* = 0.69; Figure 1B). The change in MAP (Figure 2A) and HR (Figure 2C) from baseline was averaged second by second to provide a visual of the temporal changes during 30 s of static contraction. The ΔBPi (*p* < 0.01, Figure 2B) and ΔHRi (*p* < 0.01, Figure 2D) to 30 s static contraction during the early onset stage was significantly higher in T2DM rats compared to healthy, age-matched controls. However, neither ΔBPi nor ΔHRi to static contraction were not significantly different between T2DM rats and healthy, age-matched controls during the established stage (ΔBPi: *p* = 0.67, Figure 2B; ΔHRi: *p* = 0.08; Figure 2D) or the chronic stage (ΔBPi: *p* = 0.84, Figure 2B; ΔHRi: *p* = 0.08; Figure 2D). Developed tensions to static contraction were similar for all group comparisons (Table 2, *p* > 0.05).

**TABLE 2:** Baseline MAP, HR, and TTI before muscle contraction and stretch.

Static contraction						
Stages	Early onset		Established		Chronic	
Groups	Healthy (*n* = 7)	T2DM (*n* = 8)	Healthy (*n* = 8)	T2DM (*n* = 8)	Healthy (*n* = 8)	T2DM (*n* = 10)
Baseline MAP (mmHg)	77 ± 4	82 ± 5	85 ± 5	99 ± 5	90 ± 7	89 ± 7
Baseline HR (bpm)	387 ± 16	336 ± 10*	391 ± 17	377 ± 17	368 ± 28	336 ± 15
TTI (kg.s)	19 ± 2	22 ± 1	19 ± 1	21 ± 1	22 ± 1	20 ± 1
Intermittent contraction						
Groups	Healthy (*n* = 6)	T2DM (*n* = 7)	Healthy (*n* = 8)	T2DM (*n* = 8)	Healthy (*n* = 8)	T2DM (*n* = 9)
Baseline MAP (mmHg)	74 ± 4	74 ± 4	80 ± 5	93 ± 6	79 ± 5	91 ± 6
Baseline HR (bpm)	378 ± 27	321 ± 7	407 ± 16	394 ± 23	347 ± 22	354 ± 21
TTI (kg.s)	14 ± 1	13 ± 1	12 ± 1	13 ± 1	13 ± 1	11 ± 0.3
Stretch						
Groups	Healthy (*n* = 6)	T2DM (*n* = 9)	Healthy (*n* = 8)	T2DM (*n* = 8)	Healthy (*n* = 8)	T2DM (*n* = 9)
Baseline MAP (mmHg)	74 ± 4	81 ± 5	89 ± 4	94 ± 10	86 ± 6	88 ± 7
Baseline HR (bpm)	382 ± 25	335 ± 8*	408 ± 24	358 ± 15	348 ± 20	364 ± 18
TTI (kg.s)	20 ± 0.2	20 ± 0.3	20 ± 0.2	20 ± 0.2	20 ± 0.4	20 ± 0.4

Values are means ± SE., MAP, mean arterial pressure; HR, heart rate; TTI, tension time index; Student’s two tailed *t*-test for all group comparisons within each stage. (*)significantly different from the healthy, age-matched rats, *p* < 0.05.

### Intermittent muscle contraction

**FIGURE 3:**
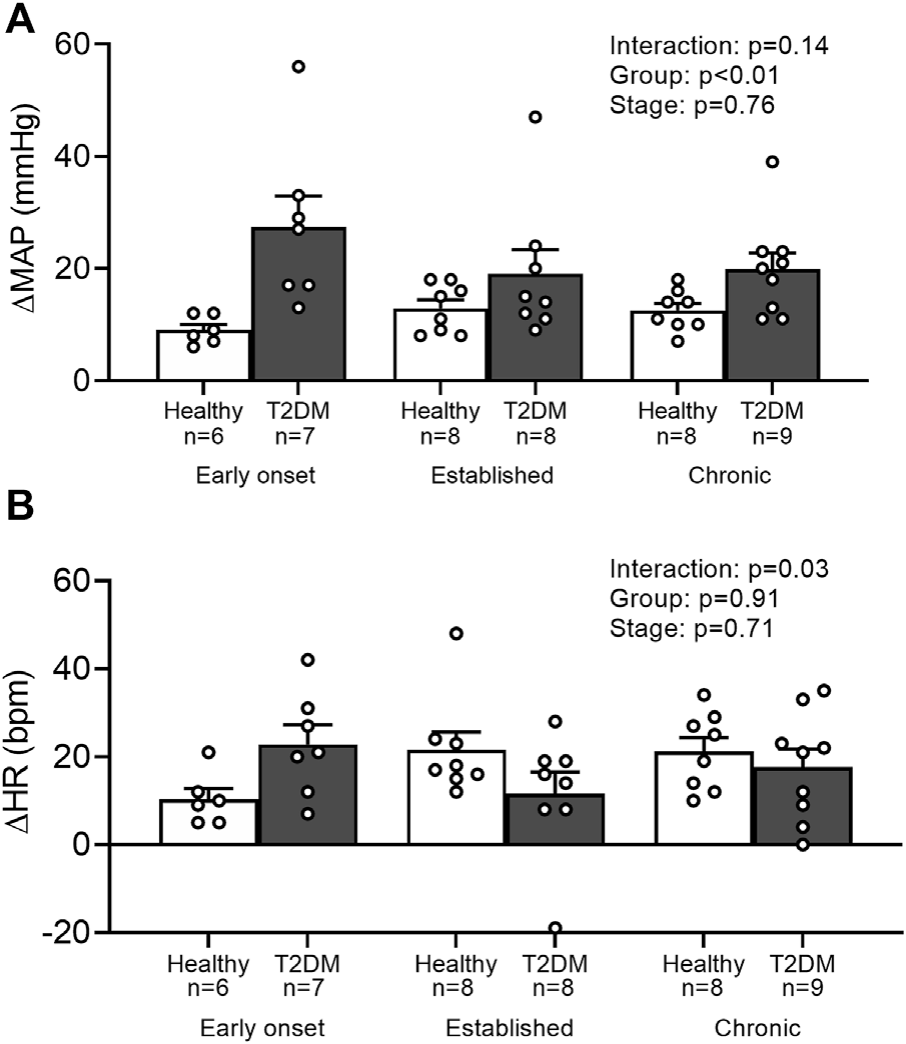
Means ± SE and individual data points showing that intermittently contracting the hindlimb muscles evoked a similar peak pressor **(A)** and cardioaccelerator responses **(B)** in male T2DM rats compared to their healthy, age-matched controls during all stages of T2DM. Two-way ANOVA with Holm-Sidak’s *post hoc* comparisons.

**FIGURE 4:**
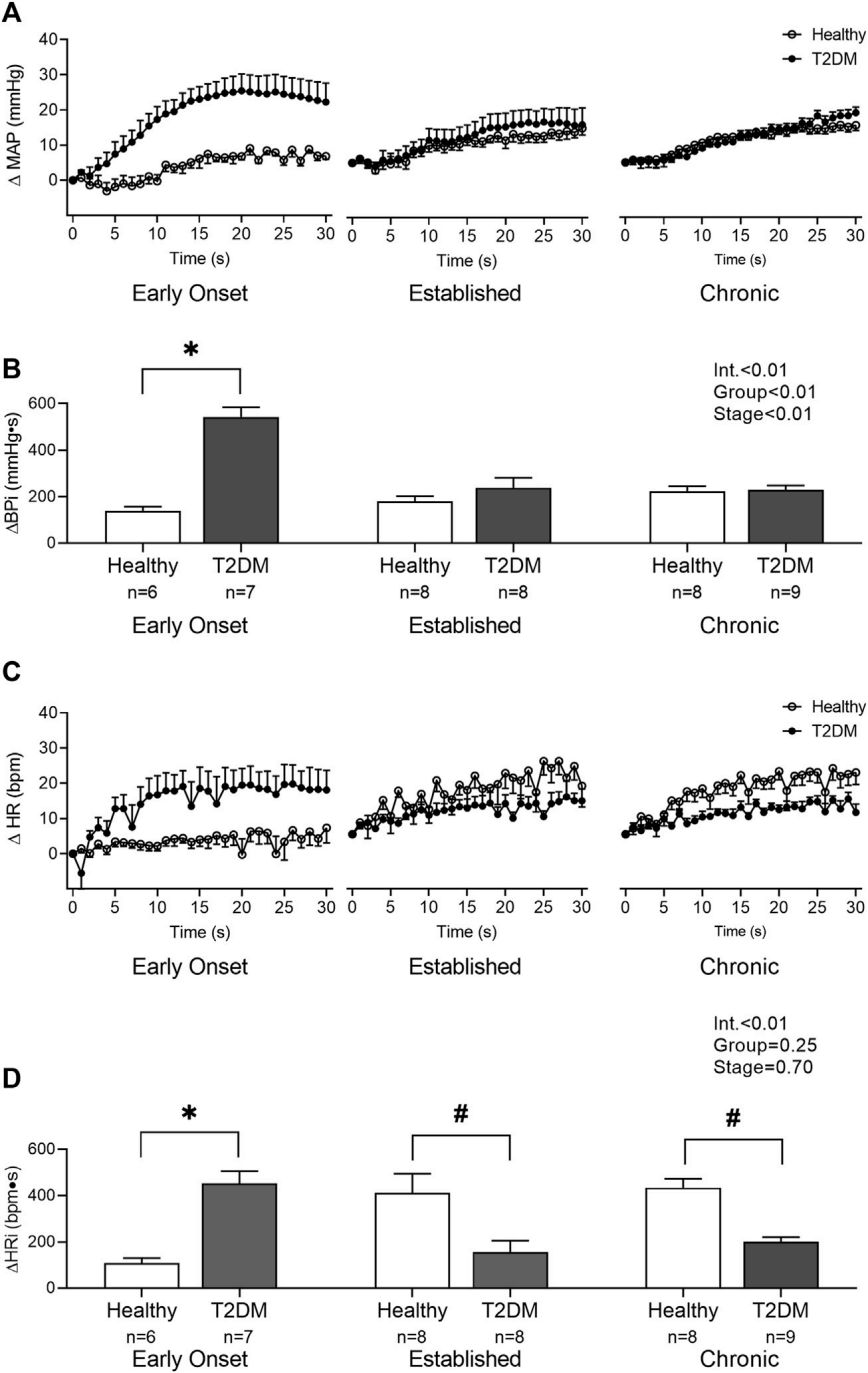
The change in MAP **(A)** and HR **(C)** from baseline were averaged second by second to show the temporal changes during 30 s of intermittent contraction in male T2DM rats and healthy, age-matched controls. ΔBPi **(B)** and ΔHRi **(D)** were then calculated over the 30 s of contraction to determine the difference between T2DM rats and healthy, age-matched controls at all stages. The ΔBPi and ΔHRi were greater in the T2DM rats compared to healthy, age-matched controls during the early onset stage of T2DM. The ΔHRi was significantly lower in the T2DM rats compared to healthy, age-matched controls during the established and chronic stage. Two-way ANOVA with Holm-Sidak’s *post hoc* comparisons (*) *p* < 0.05 indicates significant increase relative to age-matched control (#) *p* < 0.05 indicates significant decrease relative to age-matched control.

Baseline MAP and HR were similar in all groups (Table 2, *p* > 0.05). No interaction was found for the peak pressor response to intermittent contraction; however, there was a main effect of group, *p* = 0.0003. Specifically, regardless of the disease stage, the peak pressor response to intermittent contraction was significantly higher in T2DM rats compared to that in healthy, age-matched rats (Figure 3A). There was a significant interaction between group and stage for changes in HR, although no significant pairwise differences were found in the *post hoc* analyses at the early onset (*p* = 0.15), established (*p* = 0.23), and chronic stage (*p* = 0.89, Figure 3B). The change in MAP (Figure 4A) and HR (Figure 4C) from baseline was averaged second by second to provide a visual of the temporal changes during 30 s of intermittent contraction. The ΔBPi (*p* < 0.01, Figure 4B) and ΔHRi (*p* < 0.01, Figure 4D) to 30 s to intermittent contraction during the early onset stage was significantly higher in T2DM rats compared to healthy, age-matched controls. However, the ΔBPi to 30 s intermittent contraction was not significantly different in T2DM rats compared to healthy, age-matched controls during the established stage (*p* = 0.30) or the chronic stage (*p* = 0.89, Figure 4B). In addition, the ΔHRi to 30 s intermittent contraction was significantly lower in T2DM rats compared to healthy, age-matched controls during the established stage (*p* < 0.01) and the chronic stage (*p* < 0.01, Figure 4D). Developed tensions to intermittent contraction were similar for all group comparisons (Table 2, *p* > 0.05).

### Tendon stretch

**FIGURE 5:**
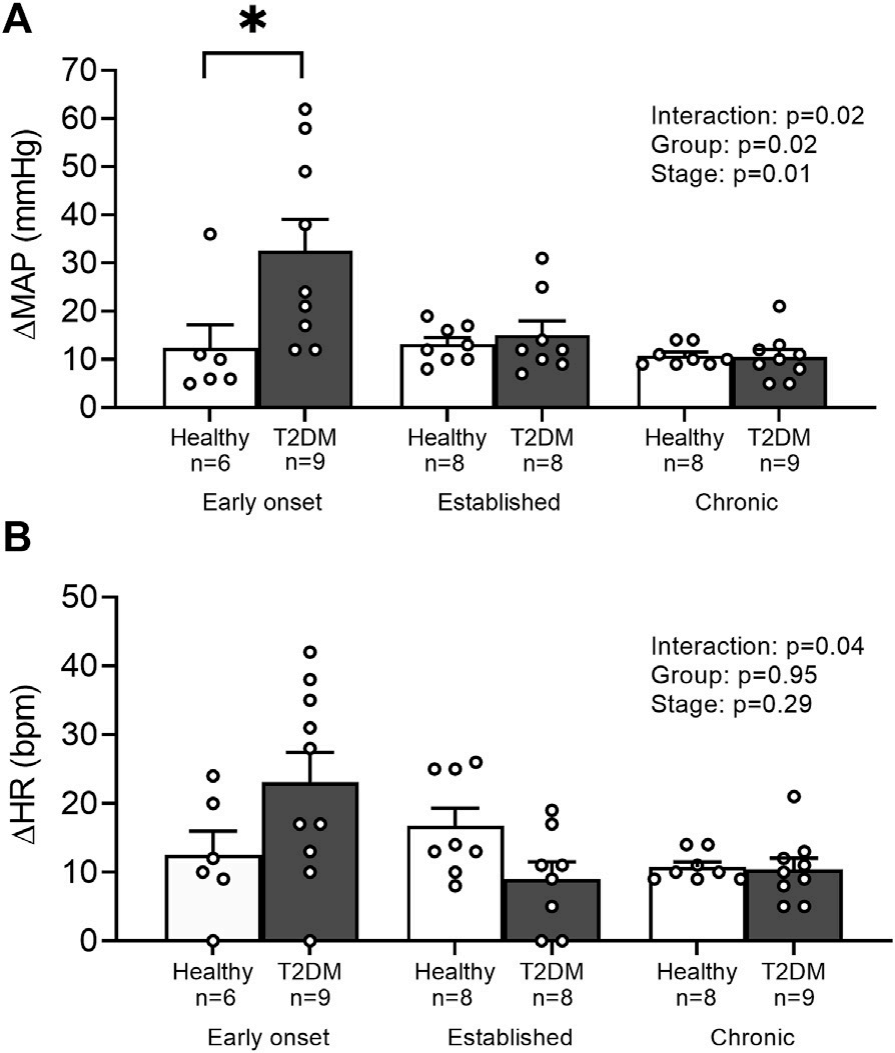
Means ± SE and individual data points showing that stretching the hindlimb muscles evoked an exaggerated peak pressor **(A)** during the early onset stage in male T2DM rats compared to healthy, age-matched controls. Cardioaccelerator responses **(B)** were similar within groups. Two-way ANOVA with Holm-Sidak’s *post hoc* comparisons (*) *p* < 0.05 indicates significant increase relative to age-matched control.

**FIGURE 6:**
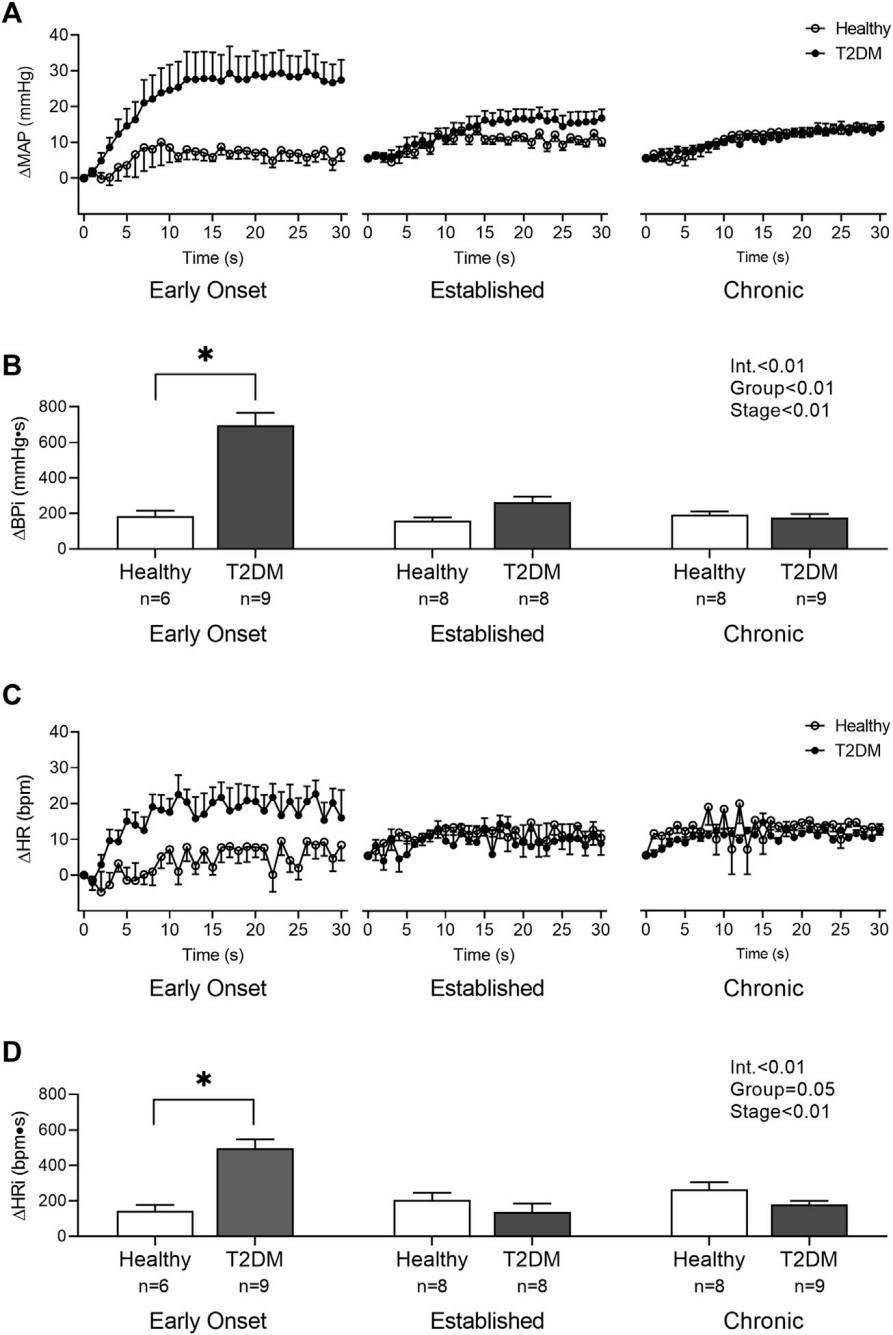
The change in MAP **(A)** and HR **(C)** from baseline were averaged second by second to show the temporal changes during 30 s of tendon stretch in male T2DM rats and healthy, age-matched controls. ΔBPi **(B)** and ΔHRi **(D)** were then calculated over the 30 s of contraction to determine the difference between T2DM rats and healthy, age-matched controls at all stages. The ΔBPi and ΔHRi were greater in the T2DM rats compared to healthy, age-matched controls during the early onset stage of T2DM. Two-way ANOVA with Holm-Sidak’s *post hoc* comparisons (*) *p* < 0.05 indicates significant increase relative to age-matched control.

Baseline MAP and HR were similar in all groups (Table 2, *p* > 0.05), except baseline HR between T2DM rats and their healthy, age-matched controls during the early onset stage, *p* = 0.04. The peak pressor response to tendon stretch was significantly higher in T2DM rats during the early onset stage compared to healthy, age-matched controls (*p* < 0.01, Figure 5A). The peak pressor responses were not significantly different in T2DM rats compared to healthy, age-matched controls during the established stage (*p* = 0.92) or the chronic stage (*p* = 0.95, Figure 5A). There was a significant interaction between group and stage for changes in HR, although no significant pairwise differences were found in the *post hoc* analyses at the early onset (*p* = 0.14), established (*p* = 0.35), and chronic stage (*p* = 0.87, Figure 5B). The change in MAP (Figure 6A) and HR (Figure 6C) from baseline was averaged second by second to provide a visual of the temporal changes during 30 s of tendon stretch. The ΔBPi (*p* < 0.01, Figure 6B) and ΔHRi (*p* < 0.01, Figure 6D) to tendon stretch during the early onset stage was significantly higher in T2DM rats compared to healthy, age-matched controls. However, the ΔBPi (Figure 6B) and ΔHRi (Figure 6D) to tendon stretch was not significantly different in T2DM rats compared to healthy, age-matched controls during the established stage (ΔBPi: *p* = 0.13, Figure 6B; ΔHRi: *p* = 0.24; Figure 6D) or the chronic stage (ΔBPi: *p* = 0.76, Figure 6B; ΔHRi: *p* = 0.24; Figure 6D). Developed tensions in response to tendon stretch were similar for all group comparisons (Table 2, *p* = 0.44).

## Discussion

The purpose of the current study was to determine the effect of T2DM on the exercise pressor reflex independent of aging. We demonstrated for the first time that the exaggerated peak pressor response to static muscle contraction and tendon stretch in T2DM rats are a result of the pathophysiology of diabetes, not aging, and are present as early as 7–15 weeks after the onset of T2DM. We also demonstrated an exaggerated peak pressor response to intermittent muscle contraction in T2DM rats; however, this response appeared independent of disease stage. Moreover, the overall increase in BP and HR throughout the entire 30 s of static muscle contraction, tendon stretch, and intermittent contraction were significantly higher in the T2DM rats compared to their healthy age-matched controls 7–15 weeks after disease onset. Therefore, our findings suggest that the exaggerated exercise pressor reflex seen in T2DM rats is due to the pathophysiology of the disease and not aging.

Aging is a major risk factor for T2DM and is known to affect cardiovascular responses to exercise. However, whether this is due to alterations in the exercise pressor reflex is inconclusive. Several studies suggest that aging may augment the BP response to exercise (Trinity et al., 2018; Hasegawa et al., 2021). Specifically, Hasegawa et al. reported that healthy older subjects had a significantly higher systolic BP response to ischemic handgrip exercise compared to that in middle-age and young subjects, and proposed increased sensitivity of the exercise pressor reflex as a possible cause (Hasegawa et al., 2021). Conversely, other studies have shown that healthy older subjects had an attenuated BP response to progressive ischemic handgrip exercise compared to that in younger subjects (Markel et al., 2003), suggesting an attenuated metaboreflex. One possible explanation for this latter response is the known age-induced metabolic shift in glycolytic to oxidative muscle fibers, which results in lower production and concentration of metabolic by-products known to stimulate the metaboreflex (Wilson et al., 1985). These conflicting results demonstrate the importance of isolating the pathophysiological effects associated with T2DM from those of aging to fully understand the sole contribution of diabetes to the exaggerated exercise pressor reflex. Similar to previous studies involving individuals with T2DM (Holwerda et al., 2016; Trinity et al., 2018), we found that T2DM augments peak pressor response, as well as the overall BP response, independent of aging. Specifically, Holwerda et al. showed that when older T2DM patients performed a voluntary isometric handgrip exercise, their pressor response was significantly higher than healthy, age-matched controls (Holwerda et al., 2016). Furthermore, we determined that the exercise pressor reflex was exaggerated 7–15 weeks after diabetes onset in this rat model that spontaneously develops the disease. Kim et al. also found that the pressor and sympathetic responses to static muscle contraction were exaggerated at a similar stage of the disease (12–16 weeks after the induction of T2DM) in streptozotocin (STZ) and high fat diet-induced T2DM rats (Kim et al., 2019). This suggests that the exercise pressor reflex is exaggerated in the early stage of the disease in both spontaneous and drug and/or diet induced T2DM. Therefore, although previous studies have shown that aging might play a role in altering the exercise pressor reflex, our finding suggests that the temporal changes of the exercise pressor reflex in T2DM occur independent of aging.

This is the first study to determine the effects of T2DM on the exercise pressor reflex and mechanoreflex independent from aging. We were surprised to find that the effects of T2DM on reflexive BP responses during exercise appear to be temporal, specifically, the pressor response was exaggerated early but not later in the disease. These temporal changes likely coincide with pathological alterations associated with T2DM. For example, it is well established that circulating insulin is upregulated in the early stages of the disease to compensate for the tissues’ inability to use insulin. Over time, however, the beta cells of the pancreas lose the ability to produce sufficient insulin, increasing the dependence on exogenous insulin and making this insulin resistant disease more similar to type 1 diabetes (Galicia-Garcia et al., 2020). Notably, a previous study from our laboratory, using STZ to destroy the insulin-producing beta cells in the pancreas, also found a similar temporal attenuation of the exaggerated pressor response to static muscle contraction (Grotle et al., 2017). Indeed, the T2DM rats in the current study did not show significantly higher insulin concentrations than healthy age-matched controls at any stages. Thus, it is possible that a shift to insulin deficiency contributes, in part, to the observed attenuation of the exaggerated exercise pressor reflex in later stages of the disease. Interestingly, these observed temporal changes from augmented to attenuated reflex responses are similar to the pathology of diabetic neuropathy. Diabetic neuropathy is a type of nerve damage that develops during the progression of T2DM (Bansal et al., 2006). With long-term insulin deficiency, diabetic patients are prone to microvascular injuries, such as epineural microvessel damage (Grover-Johnson et al., 1981; Beggs et al., 1992). The resultant damage in the neural microvasculature is associated with nerve fiber loss due to cell death from apoptosis and autophagy (Thrainsdottir et al., 2003; Malik et al., 2005; Kamiya et al., 2006). Indeed, Mehra er al. demonstrated that restoring insulin concentrations in young adult type 1 diabetic patients through pancreas transplantation improved nerve density and length, supporting the importance of insulin in preventing peripheral nerve damage (Mehra et al., 2007). Therefore, it is possible that skeletal muscle afferent nerve fibers are similarly impacted by long-term insulin deficiency, which could explain the attenuation of BP response in T2DM rats during later stages of the disease.

Although a lack of insulin causes damage to nerve fibers, excessive insulin has been shown to potentiate nerve fiber activities. Indeed, recent studies have demonstrated that both the mechanical and metabolic components of the exercise pressor reflex are potentiated by insulin (Hotta et al., 2019; Hori et al., 2022), suggesting that hyperinsulinemia may play a role in exaggerating the exercise pressor reflex in T2DM. However, in the current study we did not observe hyperinsulinemia; insulin was not significantly higher in T2DM rats compared to their age-matched healthy controls at any stage. This finding is consistent with Cummings et al., they found that plasma insulin peaked right at diabetes onset and then was significantly decreased 8 weeks later (Cummings et al., 2008). While we do not exclude that this peak in insulin earlier in the disease may still play a role, our study clearly demonstrates that the exercise pressor reflex can be exaggerated in T2DM without the concurrent presence of hyperinsulinemia. Previous studies have found that the exaggerated exercise pressor reflex coexists with hyperinsulinemia, suggesting that hyperinsulinemia must be present for an exaggerated pressor response to exercise (Scott et al., 2008; Holwerda et al., 2016; Kim et al., 2019). For example, Kim et al. reported that T2DM rats with an exaggerated exercise pressor reflex had an insulin concentration that was more than 2 times that in healthy rats (Sattar et al., 2019). In the current study, however, the insulin concentration was only half that of healthy, age-matched rats during the early onset stage, yet the exercise pressor reflex was significantly higher in T2DM rats. These findings suggest that hyperinsulinemia is not needed for an exaggerated exercise pressor reflex in T2DM and support the idea that several different mechanisms likely contribute to this altered BP response. Additional studies are needed to explore the underlying mechanisms for an exaggerated exercise pressor reflex in T2DM. Examples of potential mechanisms to explore include chronic low-grade inflammation (Copp et al., 2015; Xing et al., 2018) and/or oxidative stress (Harms et al., 2017), PKC-induced transient receptor potential vanilloid 1 (TRPV1) overactivity caused by hyperglycemia (Ishizawa et al., 2021), and changes in body weight and skeletal muscle mass and/or fiber type (Petrofsky et al., 1981; Estrada et al., 2020).

The current study is also the first to determine the effects of T2DM on the exercise pressor reflex during intermittent contraction. T2DM patients are commonly prescribed dynamic exercise as a means to manage the disease; however, previous studies have only determined the effects of T2DM on the exercise pressor reflex during static muscle contraction (Grotle et al., 2019; Kim et al., 2019). In this study, the overall increase in BP throughout the intermittent contraction was higher in T2DM compared to healthy, age-matched rats. Likewise, the overall increase in HR throughout the intermittent contraction was higher at the early onset stage in T2DM rats, but not in later stages. The exaggerated BP and HR responses increase the overall myocardial workload, contributing to potentially deleterious cardiovascular consequences in T2DM patients. These findings suggest that the exercise pressor reflex is exaggerated in T2DM regardless of the type of exercise (e.g. isometric or dynamic) evoking it.

### Clinical significance

Our findings highlight the importance of an early diagnosis of T2DM so the potential increased risk of adverse cardiovascular events during exercise is given adequate consideration when prescribing exercise interventions. Although the exercise pressor reflex does not appear to be different from that in healthy, age-matched rats in later stages of the disease, this feature is most likely not an indicator of improved cardiovascular health. Similar to diabetic peripheral neuropathy, which transitions from being expressed as hypersensitivity to hyposensitivity in response to painful stimuli, group III and IV afferents that evoke the exercise pressor reflex may be similarly losing their sensitivity as the disease progresses, resulting in attenuated cardiovascular responses during exercise. This is supported by our finding that the overall increase in HR throughout the intermittent contraction was higher at the early onset stage in T2DM rats, but significantly lower at the later stages. Thus, in addition to a heightened risk of adverse cardiovascular events in the early stage of the disease, disease pathophysiology may impair the ability to appropriately adjust the cardiovascular system to respond to the metabolic demands of exercise, especially in those with T2DM who are known for having poor peripheral circulation. Therefore, the attenuated pressor response during the later stages of the disease could ultimately result in exercise intolerance. Moreover, it is important to clarify that the findings from this study do not suggest that individuals with T2D refrain from exercising or participating in physical activities. These findings merely suggest that T2D individuals and their healthcare providers should be aware that adverse cardiovascular events may occur if participation in activities is not regulated. Further studies are needed in order to determine how best to regulate their physical activities.

In conclusion, we found that the exaggerated exercise pressor reflex seen in T2DM rats is due to the pathophysiology of the disease and not aging. Furthermore, we determined that peak pressor responses to static contraction and overall pressor responses to static and intermittent contraction were exaggerated in T2DM rats, suggesting that these responses may be observed during both isometric and dynamic exercises in T2DM patients. Therefore T2D patients’ responses to exercise could result in dangerous, acute cardiovascular events, as well as increased stress on the cardiovascular system over time, leading to poor cardiovascular health. More studies are needed to further investigate how these findings can be translated to the clinical population and how an exercise intervention can be safely prescribed to those with T2DM.

## Data Availability

The raw data supporting the conclusion of this article will be made available by the authors, without undue reservation.
